# Using the Guttman Scale to Define and Estimate Measurement Error in Items over Time: The Case of Cognitive Decline and the Meaning of “Points Lost”

**DOI:** 10.1371/journal.pone.0030019

**Published:** 2012-02-17

**Authors:** Rochelle E. Tractenberg, Futoshi Yumoto, Paul S. Aisen, Jeffrey A. Kaye, Robert J. Mislevy

**Affiliations:** 1 Departments of Neurology, Biostatistics, Bioinformatics & Biomathematics, and Psychiatry, Georgetown University Medical Center, Washington, District of Columbia, United States of America; 2 Collaborative for Research on Outcomes and –Metrics, United States of America; 3 University of Maryland University College, College Park, Maryland, United States of America; 4 Department of Neurology, University of California San Diego, La Jolla, California, United States of America; 5 Layton Alzheimer's Disease Research Center, Oregon Health Sciences University, Portland, Oregon, United States of America; 6 Educational Testing Service, Princeton, New Jersey, United States of America; McGill University/Douglas Mental Health Univ. Institute, Canada

## Abstract

We used a Guttman model to represent responses to test items over time as an approximation of what is often referred to as “points lost” in studies of cognitive decline or interventions. To capture this meaning of “point loss”, over four successive assessments, we assumed that once an item is incorrect, it cannot be correct at a later visit. If the loss of a point represents actual decline, then *failure* of an item to fit the Guttman model over time can be considered measurement error. This representation and definition of measurement error also permits testing the hypotheses that measurement error is constant for items in a test, and that error is independent of “true score”, which are two key consequences of the definition of “measurement error” –and thereby, reliability- under Classical Test Theory. We tested the hypotheses by fitting our model to, and comparing our results from, four consecutive annual evaluations in three groups of elderly persons: a) cognitively normal (NC, N = 149); b) diagnosed with possible or probable AD (N = 78); and c) cognitively normal initially and a later diagnosis of AD (converters, N = 133). Of 16 items that converged, error-free measurement of “cognitive loss” was observed for 10 items in NC, eight in converters, and two in AD. We found that measurement error, as we defined it, was inconsistent over time *and* across cognitive functioning levels, violating the theory underlying reliability and other psychometric characteristics, and key regression assumptions.

## Introduction

Acknowledging and understanding the error associated with measurement is crucial to improving statistical modeling. Commonly, independent variables are treated as if they are error-free, with responses independent over time [1]; error-free independent variables is a key assumption of regression [2]. Measurement error is a source of variability that has traditionally *not* been considered in neuropsychology, including the study of cognitive aging or Alzheimer's disease (AD) (although see [3] and [4] for counter-examples). Under classical test theory (CTT; see [5], [6]) observed scores (e.g., cognitive or personality test scores) are considered imperfect representations of the ‘true’ construct in which we are actually interested. Intra-individual variability (IIV) can play a significant role in the design, analysis and interpretation of psychological and cognitive outcomes (see [4]); in cases where investigators want to utilize IIV as a longitudinal outcome, rather than change in total scores, teasing the variability apart from extent to which a test fails to reflect what is targeted (“real” error) is especially important.

Typically, clinical studies of, and trials of interventions to affect, AD and mild cognitive impairment are powered to detect a minimum number of “points lost” – representing cognitive decline. Although clinicians do not necessarily believe that once a point on any cognitive test is lost the capacity to answer correctly itself is permanently lost, the number of points “lost” is used to represent the amount of cognitive decline that was observed and/or prevented (e.g., [7]–[13]; see also [14]).

CTT defines the observed score X as a function of some “true” but unobservable score *T* plus some “error” that is specific to the individual (X = *T*+*e*) [5]. The true score for an individual is an unknown constant and the error with which this true score is measured (yielding X) is an unknown random variable, defined as being independent of the true score. While the “true score” does not represent “The Truth” in an absolute sense, it does represent the error-free version of an individual's test performance under CTT. This definition implies that the test's error will not vary systematically, irrespective of the true score.

Recent work has shown that *reliability* in cognitive variables *can* vary within individuals [4]. Since reliability can be estimated under CTT as 1-error, this work suggests that assuming a constant error for any given test might not be appropriate - although this is a consequence when psychometric characteristics are derived under classical test theory. The ability to test the independence of measurement error and true score would be useful for investigators who use “high reliability” or “low measurement error” as a criterion for choosing a test.

If the definitions of error and true score under CTT *do* hold, then a reliability coefficient for any given test can be calculated and interpreted, and measurement “error” can be estimated as (1-reliability) (among other formulae; see [15], pp 69–70; [16]). If the CTT definitions do *not* hold, more complex theoretical and modeling approaches to reliability are available (see [17]; see also [5] and [6]), although these models are not widely used outside of formal psychometric contexts (although see [18] for a new application of modern/formal measurement theory to widely available tests for clinical research).

“Reliability” under CTT is a widely used construct across many disciplines, but to compute and interpret it assumes that the distribution of error associated with a test is identical for all respondents and that the error is independent of the respondent's true score. However, X = *T*+*e* is not a model, it is a definition ([5], pp. 119–123); this paper describes a method to define measurement error so as to test these implications – because they are *not testable* under CTT ([5]; pp 119–123; [15]; pp 68–9). Our definition of measurement error is based on the assumption that “point loss” corresponds to “cognitive decline”. This restrictive assumption is consistent with the use of the conceptualization of a total score over time representing an individual's level of cognitive functioning (e.g., [7]–[13]). This is the first definition of measurement error that can be studied empirically. We use this definition and approach to estimate measurement error in groups whose “true scores” differ in this study. Comparing error estimated under our method across these groups will permit us to empirically test the CTT-derived hypotheses that error is independent of true scores and that it is constant for a test.

Our model of measurement error is an adaptation of the Guttman Scale [19]. A key property of a Guttman Scale is that for any set of items, there is a single hierarchy of endorsement, acquisition (or loss), or preference. That is, for a set of ordered items that fit a Guttman Scale, if later items are correct or endorsed, then it is assumed that all earlier/easier/prerequisite items are correct or endorsed as well. Thus, every person with a given total score will have the same pattern of responses [20]–[23]. This is not an explicit assumption of any cognitive tests in clinical use today. It is, however, *consistent* with the definition of “cognitive decline” based on observing that points on any cognitive test have been lost over time, and is also implied by the use of these terms in common practice [6]–[13], [24]–[27].

Under our approach, responses to one item over time are treated as the “hierarchy”. Each item is individually modeled as a unidimensional measurement of the ability to respond to that item over successive evaluations. In our Guttman model of a cognitive test item *over time*, correct answers at later visits imply that the item was correctly answered at all previous visits. An incorrect answer at a visit implies that the item was (will be) incorrectly answered at all successive visits; nothing is implied about previous visits. This model represents a literal “cognitive loss” in the sense that an incorrect answer is assumed to reflect the loss of the ability to respond correctly. The main difference between our approach and a standard cross-sectional Guttman approach is that we have defined “measurement error” for a given item as a failure of that item over time to fit a Guttman model. That is, “error” in any item is defined as a failure of the item to provide a consistent “signal” about the individual's cognitive state over successive evaluations (a model of a definition of reliability given in [28], p. 277]). “Consistency” is defined as observing a pattern for a given item over time that is consistent with the Guttman model (see [29]). Crucially, this approach does not distinguish patterns that are inconsistent with the Guttman model are observed because of actual measurement error as we have defined it (“systematic error” [29]) from those due to an error that was not a function of the item (“random error” [29]). The Mini Mental State Exam (MMSE, [30]) is commonly used to test cognitive functioning, and like most cognitive instruments it is a combination of items that were selected to represent different cognitive abilities. Tests such as the MMSE are multi-dimensional, complicating the estimation of reliability and measurement error. Further, because cognitive tests such as the MMSE are not all useful across the full dementia severity range (see, e.g., [14], Ch. 18), it is an excellent representative on which to test our measurement error definition.

## Methods

### Ethics Statement

Existing data, collected with informed consent from the subjects under federally-funded projects at Oregon Health & Science University (OHSU), were shared with the first author in accordance with the policy of the National Institutes of Health (NIH) encouraging data sharing (see http://grants.nih.gov/grants/policy/data_sharing/), all relevant federal (USA) data sharing and personal data protection regulations, and OHSU-specific requirements (see http://www.ohsu.edu/xd/research/centers-institutes/neurology/alzheimers/research/data-tissue/data-use-policy.cfm). All individuals in the data set were administered the same set of instruments at OHSU by trained and experienced personnel under NIH-funded, IRB-approved protocols, after obtaining written consent specific to the study in which their data were collected and the ongoing maintenance of a database (as outlined in the above-listed URL). None of these studies were clinical trials. Only the first four years' (of a possible 16) of any participants' visits were modeled so as to capture sufficient time for cognitive changes (and errors in their detection) to be observed, while not excessively limiting the sample size. The data analyzed in this study includes all participants (described below) whose data were archived as of November 2004.

### Participants

**Subjects with AD.** Subjects with AD (“AD”, N = 329) are patients from the Aging and Alzheimer's Clinic (the clinical core of the NIA-funded Layton Alzheimer's Disease Research Center at OHSU). They originally presented with memory complaints, either on referral by self, family or health care provider. On enrolling in the OHSU registry for participation in an NIA-sponsored longitudinal study, each subject's clinical history and exam findings were presented at a weekly case conference where a consensus diagnosis (based on standard criteria at the time [31]) was reached by the neurologists, geriatric psychiatrists, neuropsychologists and research nurses of the OHSU Alzheimer's Disease Center. A battery of tests was administered on each annual visit, according to the protocol. Of the 329 patients with data, 78 had MMSE item level information at their first four successive visits.

**Non-demented elderly subjects.** Cognitively intact participants (“NC”, N = 412) are research subjects of the Oregon Brain Aging Study (OBAS [32]–[33]), a federally-funded (US Veterans Affairs and National Institute on Aging, NIA) project to study normal neurological aging. These subjects were known to be cognitively intact based on the extensive neurological and neuropsychological assessment they received on enrollment in OBAS, and on each successive annual evaluation. Of the 412 persons with data, 149 had MMSE item level information at their first four successive visits.

**Subjects with subsequent “other” or questionable/incipient dementia diagnoses.** Subjects in the “AD to be” cohort (“Converters” N = 185) are individuals from the Aging and Alzheimer's Center Clinical Core who were found to be cognitively intact (i.e., enrolled in OBAS) at their first visit to the clinic and who subsequently were diagnosed with questionable, possible or probable AD. On their first visit, the clinical history and exam findings for each person in this group were presented at a weekly case conference where a consensus diagnosis – that the patient did NOT meet diagnostic criteria for possible or probable AD [31] - was reached by the clinical team. However, at a follow up annual visit, the individual was characterized as no longer meeting the criteria for non-demented elderly. Of the 185 persons with data, 133 had MMSE item level information at their first four successive visits.

### Instrument

The Mini-Mental State Examination (MMSE [30]) is a 30-point test with items requiring attention, orientation, calculation, memory, language, and visuospatial functioning. The MMSE, and change on it, has been used as an outcome measure in clinical studies, but it is also prevalent as an inclusion criterion for clinical studies and clinical trials in AD.

### Data Analysis

These analyses focused on whether each item over four years fits the Guttman model in each of three cohorts modeled separately. To the extent that the item does fit the model, it represents within-person consistency with a “cognitive loss” interpretation of a change from correct to incorrect response over successive visits (and a “cognitive stability” interpretation of the same answer at any two successive visits). We characterized deviations from this assumption as “measurement error” (ME, described below) and compared these estimates across items and cohorts. If ME is not different for items or cohorts, then standard reliability coefficients can be computed and interpreted. If ME differs for items, or cohorts, then key assumptions for regression (error free independent variables) *and* key CTT implications are violated, so that standard reliability coefficients cannot be interpreted.

### Scoring MMSE items

The MMSE has 11 items worth a total of 30 points (using the scoring given by [34]). The data for two items, worth 3 points each (name 3 items and follow 3-stage instructions), were not entered into the data file in a manner that could be consistently recoded to the 0/1 required by a Guttman model, so these items were unmodeled. Responses on three items (WORLD spelled backwards, 3-item recall, and repeat ‘no ifs, ands or buts’) were recoded (unless missing) so that perfect performance was ‘correct’ (1) and otherwise, responses were recoded as 0. Two items (“what county are we in?” and “what hospital are we in?”) could not be modeled because they had high proportions of missing responses due to changes over time in which question was used, while insufficient variability was observed in two additional items (take this paper, fold it in half) so that models did not converge. We assigned one point to *each* of the two naming items (typically one point is allotted for the two correctly-named items). Thus, nine of the original 11 MMSE items were modeled (giving a total of 16 points). These manipulations of the item-level data were data driven, and not theoretically motivated – in keeping with our objective that this method be usable beyond the assessment of cognitive decline.

### The Guttman model of measurement error over time

Table 1 reflects a Guttman model of one item over four visits. Over four successive visits, if an item is incorrect (“0”) at one evaluation, it should not be correct (“1”) at a later visit, or else the item is not consistent with the Guttman model. Labeling incorrect answers as zeros and correct answers as ones, four zeros and ones represents performance on a single item over four test sessions (annually in this context).

**Table 1:** Example Guttman Scale response patterns for one item over four visits.

Observed response pattern on one item	Time 1	Time 2	Time 3	Time 4
Pattern 1	1	1	1	1
Pattern 2	1	1	1	0
Pattern 3	1	1	0	0
Pattern 4	1	0	0	0
Pattern 5	0	0	0	0
Pattern 1 of 11*	0	1	0	0

Notes: 1 indicates the item was answered correctly; 0 indicates it was incorrect. Patterns in the first five rows are consistent with the Guttman scale. NB: the first and fifth patterns (1111, 0000) do not represent *decline* since individuals with either pattern of responses to this item over the four visits either always or never exhibited the ability to answer correctly (respectively). Both patterns **are** consistent with a Guttman Model because each shows the expected consistency in what an item reflects about the individual's state/ability.

*indicates one example pattern of the 11 other possible outcomes for one item over four visits; none of these other patterns is consistent with a Guttman Model since the item is shown to have been correct after not being correct at an earlier visit. There are a total of 16 (2^4^) patterns of right (1) and wrong (0) responses on this item, but only the first five response patterns in this table represent error-free measurement of decline for the item. The proportion of the sample that does not exhibit one of these five patterns over four years is the estimated measurement error for the item.

An item can be correct (1111), or incorrect (0000), at all visits and still be consistent with this model. Both of these patterns would represent “stability” over time, and this is critical for interpretation of a reliability coefficient, i.e., that it gives the same information over repeated assessments. We defined measurement error as a pattern of responses to an item over time that is inconsistent with the Guttman model. The proportion of the cohort with inconsistent patterns on an item represents the estimated measurement error for that item for that cohort.

### Model fit and measurement error

Modeling proceeded using parameters and coding developed by Dayton [22]–[23], outlined in Appendix (see also [35]–[36]) for Excel (2003, Microsoft Inc., Redmond Washington). Model fit for each of the MMSE items was summarized with two statistics. The first, π* (“pi star”; [37]; see also [22]–[23]), is an index of how ‘far’ from a perfect fit to the data the model is [22]–[23]. The value of π* indicates what percent of the observations would need to be eliminated to achieve perfect fit of the given model for that item. We used π* to estimate the ‘level’ of measurement error for each of the items. The associated standard errors of the π* values were also estimated [36]. There is no inference test associated with this index; values<0.10 are typically used to indicate acceptably small differences between observed and expected frequencies [22].

An additional summary statistic is the dissimilarity index (DI [22], [37]), which compares expected and observed frequencies of patterns for the set, based on the assumed model. Large DI values suggest that the pattern frequencies expected, given that the model is true, are “extremely different” from the observed frequencies. There is no inference test associated with this index; values<0.05 are typically used to indicate acceptably small differences between observed and expected frequencies [22]–[23].

For both indices, low values suggest better fit of the model to the data; we could have constructed likelihood ratio tests or computed information criteria to facilitate inference testing or comparisons of our model (fully constrained) against less-constrained models, but our objective was to count the number of items in each group that did and did not fit the Guttman model. In cases where only one pattern was observed for an item, computations of these fit statistics cannot converge, providing no information about measurement error for that item. We calculated π* and DI for all MMSE items for which <5% of responses were missing, the fit index would converge, *and* that we could score as 1/0. Since π* has an associated SE and an interpretation consistent with our objective, this was our main outcome.

### Method

For each item scored as 0/1, a “response vector” for each participant was constructed using responses obtained over four years. The first four annual visits were chosen to maximize the sample size (i.e., the number in each group with multiple consecutive visits) while also capturing a time frame within which cognitive changes might be observable and detectable. Sample sizes dropped precipitously in all cohorts after the fourth year.

Table 1 presents those five vectors of responses on a single item over four years that correspond to the Guttman model of change, i.e., only these five patterns should be observed if an item can be considered to be indicating “real loss” (or “real stability”, 0000 and 1111). There are 16 possible vectors (2^4^) with which an individual could respond to an item scored 0 (wrong) or 1 (right) over four time points. The proportion of each group exhibiting each of the 16 possible response vectors was calculated per item with Excel [22]–[23], and the π* and dissimilarity index values were computed based on a five-class restricted latent class model [23], [38] (see modeling and estimation details in Appendix S1; modeling code is available by request from Dr. Yumoto). Values were estimated for each group, as well as over all individuals.

## Results

General descriptive statistics for the three groups are presented in Table 2. We did not compare the groups statistically on any demographic variable since neither similarities nor differences in the groups were relevant to our analysis. We also did not explore co-morbidities in terms of psychiatric diagnoses since none of the study participants had such evaluations.

**Table 2:** Descriptive Statistics (% or Mean (SD)) for three cohorts of elderly MMSE respondents with four consecutive visits.

	NC (N = 149)	Converters (N = 133)	AD (N = 78)
Age (Time 1)	83.6 (6.7)	84.3 (6.9)	70.8 (9.3)
% Female	63%	62%	46%
Education (yrs)	13.9 (2.7)	14.0 (2.8)	13.7 (3.3)
MMSE Total: Time 1	28.6 (1.3)	27.8 (1.7)	22.2 (4.6)
MMSE Total: Time 2	28.4 (1.3)	27.8 (1.8)	20.9 (5.6)
MMSE Total: Time 3	28.4 (1.3)	27.4 (2.2)	17.8 (6.8)
MMSE Total: Time 4	28.6 (1.3)	27.1 (2.6)	14.5 (7.7)
MMSE 16 items: Time 1	14.9 (0.9)	14.4 (1.2)	11.2 (2.6)
MMSE 16 items: Time 2	14.7 (1.0)	14.4 (1.3)	10.6 (3.1)
MMSE 16 items: Time 3	14.8 (1.0)	14.3 (1.4)	8.8 (3.7)
MMSE 16 items: Time 4	14.8 (1.1)	14.1 (1.6)	7.0 (4.1)

MMSE Total: range from 0–30. MMSE 16 items: sum of 0/1 score on the 16 items shown in Tables 3 and 4.

The patients tended to be younger (mean age 70.8, SD: 9.3 years) than the non-demented elderly (mean age: 83.6, SD: 6.7 years) as well as those who were initially cognitively normal but who were later diagnosed as having some cognitive impairment (mean age: 84.3, SD: 6.9 years). The patient group was 46% female while the other two groups were less balanced (NC: 59% female, Converters: 62% female). MMSE total scores for the four visits are included in Table 2 for reference; not surprisingly the two non-demented groups (at baseline) had very similar total MMSE scores while the patient average was lower.

### Model fit results

DI values of <0.05 indicate small differences (5%) between what was expected given the model and what was observed, and π* values of .10 or higher suggest that 10% or more of the data for that item would need to be eliminated to obtain perfect fit of the model to the data for that item [22]–[23]. We focused on π* values, because they offer estimated standard errors, and used the 0.10 value as a rule of thumb for interpretation of fit results. DI values were computed as ancillary summary information. Table 3 presents the π* values and Table 4 presents the DI values that could be calculated per item, for the three groups separately, as well as the overall values. The overall values were included to highlight whether any overall measurement error could be traced to one or another group or could be considered ‘inherent’ to the item itself.

**Table 3:** π* statistics (standard error), reflecting badness of fit of a Guttman model to each modeled MMSE item over four years.

MMSE Item	Over All Groups	NC	Converters	AD
Year	**0.030 (.009)**	**0.007** (.007)	**0.015** (.011)	0.103 (.035)
Season	0.164 (.020)	0.101 (.025)	0.152 (.031)	0.308 (.053)
Date	0.269 (.039)	0.235 (.035)	0.152 (.031)	0.231 (.041)
Day	0.103 (.016)	**0.040** (.016)	**0.068** (.022)	0.282 (.052)
Month	**0.075 (.014)**	**0.020** (.012)	**0.045** (.018)	0.231 (.048)
State	**0.017 (.007)**	**0.000**	**0.000**	**0.077** (.031)
City	**0.036 (.010)**	**0.000**	**0.023** (.013)	0.128 (.038)
WORLD†	0.264 (.025)	0.241 (.036)	0.299 (.043)	0.245 (.063)
3 word recall†	0.431 (.026)	0.403 (.090)	0.451 (.043)	**0.077** (.031)
Paper on floor	0.144 (.019)	**0.034** (.015)	0.158 (.032)	0.333 (.054)
Pencil§	**0.008 (.005)**	**0.000**	**0.000**	**0.041** (.023)
Watch§	**0.004 (.027)**	**0.000**	**0.000**	**0.014** (.014)
No ifs/ands/buts†	0.302 (.024)	0.302 (.038)	0.371 (.042)	0.182 (.045)
Read	**0.039 (.010)**	**0.027** (.013)	**0.030** (.015)	**0.077** (.031)
Write	**0.064 (.013)**	**0.034** (.015)	**0.030** (.015)	0.179 (.044)
Copy	0.248 (.023)	0.255 (.036)	0.278 (.039)	0.182 (.045)

π* estimates the proportion of observations that are inconsistent with the model under investigation. Low values of π* suggest that very little (100%×π*) of the data do not fit the model under investigation. Bold values of π* indicate acceptably LOW (<10%) levels of misfit; that is, bold values indicate consistency of the item with the Guttman (‘real’ loss) Model.

† This item was recoded so that all possible points right = 1 and any mistakes = 0.

§ These items were each assigned one point (i.e., not treated as one point together). Items not represented in this table did not have 0/1 coding (*name 3 items*), had too much missing data (*what floor are we on*? *What county are we in*?) or failed to converge (*take this paper*, *fold it in half*) in all 3 groups (and over all responses) so estimates of π* were not computable.

**Table 4:** Dissimilarity Index (DI) values per item, over all participants and separately by cohort (* indicates that the solution for DI did not converge so no index value was calculated).

MMSE Item	ALL	NC	Converters	AD
Year	**0.029**	*	*	0.077
Season	0.072	**0.013**	0.065	0.123
Date	**0.038**	0.120	0.067	0.110
Day	0.059	*	**0.034**	0.125
Month	0.068	*	**0.030**	0.131
State	**0.016**	*	*	0.056
City	0.079	*	*	0.100
WORLD†	0.111	0.093	0.150	0.125
3 word recall†	0.144	0.119	0.088	**0.051**
Paper on floor	0.084	**0.007**	0.074	0.109
Pencil§	**0.008**	*	*	**0.037**
Watch§	**0.002**	*	*	**0.013**
No ifs/ands/buts†	0.060	0.121	0.103	0.084
Read	**0.024**	*	*	**0.044**
Write	**0.040**	*	**0.029**	0.275
Copy	**0.052**	0.060	0.136	0.150

Note: Dissimilarity indices computed for each item represent how well the model under investigation produces expected distributions of response patterns (e.g., from Table 1) that are consistent with observed response patterns for each item. Higher values suggest less consistency between observed and expected values; one recommended cutoff for the index is 0.05 (Dayton, 1998) but this is essentially an arbitrary index value cutoff. Bold items have values below 0.055.

*indicates additional convergence problems when DI was computed.

† This item was recoded so that all possible points right = 1 and any mistakes = 0.

§ These items were each assigned one point (i.e., not treated as one point together). Items not represented in this table did not have 0/1 coding (*name 3 items*), had too much missing data (*what floor are we on*? *What county are we in*?) or failed to converge (*take this paper*, *fold it in half*) in all 3 groups (and over all responses) so estimates of π* were not computable.

Collapsing across all respondents, of the sixteen items that we could model, the π* values for six items met our criteria for “fit by a Guttman model”, i.e., could be considered to reflect loss without appreciable error (Table 3). These items were to give the year, name the state and city, spell WORLD backwards, name pencil, name watch, and read to command (all π*<.05). In fact, 7.5% or less of the full dataset would need to be eliminated for perfect fit of these items, plus naming the month (π* = 0.064) and writing to command (π* = 0.075), to a Guttman model. Between 10% (name day) and 43% (‘3 word recall’, recoded as 0/1) of the dataset would need to be eliminated for a perfect fit in the other modeled items. In terms of DI over all respondents (Table 4), give the year, date, and state, name a pencil or watch, and read, write and copy to command all had DI<0.05. Another four items (name the season, day, and month, and repeat ‘no ifs, ands or buts’, recoded as 0/1) had DI<0.075.

For the non-demented elderly controls, ten items (year, day, month, state, city, paper on floor, name pencil, name watch, read, write) met our π* criterion for error-free measurement of loss (or in their case, stability) over time (π*<0.10). The six items *no*t meeting the criterion for error free measurement reflected from 10.1% (name season) to 40% (3-item recall, recoded as 0/1) measurement error. There was very little loss in this cohort over four years in the average of either the total MMSE score or the sum of the 16 items fit with the Guttman model. This homogeneity (high proportions of items correct at all visits) is reflected in the failures of all but seven items to converge to a DI (Table 4). Of the seven DI that were calculable, five failed to meet a 0.05 cutoff (one of these (copy to command) had DI<0.075). The two items with DI<0.05 were name the season and put paper on the floor.

For those who were initially non-demented but later were diagnosed with a cognitive impairment, nine of the 16 items with converging calculations gave error-free measurement of loss over time in this cohort according to π* (year, day, month, state, city, name pencil, name watch, read, write). The seven items that *failed* to meet the π* criterion for error-free measurement of loss over time (season; date; WORLD backwards, 3-word recall, paper on floor, no ifs ands or buts; copy design) reflected between 15% and 45% measurement error. Similar to the case with the control group, there was very little change over time in this cohort and DI (Table 4) failed to converge for six of the 16 items. Of the eight DI that were calculable, three were under 0.05 (three others (season, date, put paper on floor) having DI<0.075).

For the AD patients, five of the 16 items (state, 3 word recall, name pencil, name watch, read) met our π*<0.10 criterion for error-free measurement of loss over time. For the 11 other items that failed to meet the definition of ‘error free’ over time, error was estimated to range between 10% and 33%. All of the 16 items had convergent dissimilarity indices for this cohort (Table 4), and of these, three had DI<0.05 (pencil, watch, read); two additional items (3 word recall and name the state) had DI<0.075.

## Discussion

We defined measurement error assuming only that the same item, administered annually, requires the same trait(s) for correct response, such that an incorrect response implies the loss of the trait. This is not especially realistic, but reflects clinical expectation of what the items are ‘measuring’ and how this is expected to change over time (e.g., [7]–[13]; [24]–[27]), although our method does not distinguish “systematic” and “random” error types [29]).

We found that most (10/16) of the MMSE items over four visits were consistent with our model for the control group, and that fewer items over the same time span were consistent with the Guttman model for the other two groups. This suggests that measurement error, as we defined it, depends on the level of the underlying construct; it was also different by MMSE item.

This definition of measurement error as a “signal” about change over time empirically estimable; and our results do not support the selection of cognitive tests using CTT-derived estimates of reliability and measurement error. Additionally, the results do not support the assumption that the MMSE is an error-free independent variable in regression. In contexts where point loss on tests like the MMSE and cognitive decline are equated (e.g., [7]–[13]; [24]–[27]), standard regression analyses, as well as typical reliability coefficients, may not provide the expected information (see [28]–[29] for discussion of limitations of reliability for variables that change over time). Because this method considers one item at a time, the method could be useful for unidimensional *and* multidimensional instruments.

There are many limitations to this study. Firstly, it is possible that some MMSE items **do** reflect state-based ‘cognitive loss’, while others do not; our results do not address whether any of the items that we could *not* fit are of this state-based loss type. We were able to evaluate (i.e., generate converging models and their estimates for) 16 of 30 points on this test, so even if all the other items passed our definition of “error-free measurement over time”, which we could not establish, the test as a whole would still be inconsistent with the CTT-based reliability coefficient.

We also treated several items (3-item recall and WORLD spelled backwards, repeat “no ifs, ands or buts”) as dichotomous (all right/all wrong). This facilitated the interpretability of our definition of measurement error for these items – but a more complete evaluation of these – and other- polytomous items, including a sensitivity analysis to determine if our approach yields different error rate estimates depending on scoring, will be an important future study. Also, several items exhibited too little variability within a group to estimate our summary statistics. That is, for any item where all respondents exhibited the same response pattern over time, even if it was consistent with the Guttman scale, that would be insufficient variability for the model to converge. Validating our definition of measurement error in a new sample would be an ideal context for exploring the specific item and item-type performances.

Our model *implies* conditional independence [20], [22]–[3] because we modeled each item as requiring one skill over time. Therefore, when the effects of that skill are conditioned on, the response likelihoods become random. There might be some residual memory for the item over time, but this should be minimal because the test is just one in a large battery, and the assessments are 12 months apart. In cases of residual dependency, it *could* be attributed to memory for the item, and so would be expected to decrease as the respondent's cognitive impairment increases, and might have contributed to our observation of more items failing to fit the Guttman model as cohort impairment increased. Therefore, it is possible that some of the increase in numbers of items failing to fit the Guttman model as cognitive impairment increased might be attributable to decreasing memory for the item over time. This is typically *not* taken into consideration in clinical applications where “point loss” is equated with “cognitive decline”, and it is unlikely that this explains *all* of our results.

We were unable to test whether depression, anxiety, or other comorbidities may have differentially affected either item-level performance, performance by each of the diagnostic groups we studied, or other aspects of our definition of “measurement error”. We were also unable to integrate item-level covariate information, such as varying sensitivities of individual MMSE items to comorbidities, particularly if these might vary over disease severity, the presence of mixed dementias or cerebrovascular features, age, sex or educational attainment by the study participants.

A final limitation is that our study required as large of a sample, with item-level data, as possible, and sufficient time to, for example, ensure that the cognitive normal controls were normal throughout their observation period (1–16 years), and to observe transitions in participants who entered the observational study with a consensus “diagnosis” of cognitively normal and achieve a clinical diagnosis at a later visit. Balancing these requirements led to our focus on the first four successive evaluations – and also to considerable decrement in our samples. Future work to support any generalizations of our results will also need to address the different attrition rates in our three groups.

By applying the label “measurement error” to failures of patterns of responses on items to fit the Guttman model, and comparing error rates across items and our three diagnostic samples, we tested the hypothesis that measurement error was independent of “true score” for the first time in the cognitive assessment domain. We chose the Guttman model *because* it is highly restrictive, and because it maps to the use – if not the intention- of the construct of “point loss” representing cognitive decline. Less restrictive definitions of “error” might lead to more consistent error rates across severity (“true score”) levels. Future work could explore our definition compared to others (including other models, such as [28]) across multiple samples. The method can easily be adapted for estimating measurement error in other instruments or disease populations, so that the interpretability of psychometric characteristics (particularly those derived from CTT) in those contexts can also be studied. If, as we found, the evidence suggests that CTT definitions for interpretable reliability estimates are not supported, alternative estimation – or selection criteria - should be used.

The “10% rule” as our π* cutoff represents a willingness to accept up to 10% of misfit, which could include increasing variation or recovery. Our method provides no information about the sensitivity to, or reliability for estimating, fluctuating performance (e.g., [39]), although importantly, current usage of tests such as the MMSE is almost exclusively to detect “cognitive decline”. CTT-based reliability estimates are often used to choose the tests to be employed as inclusion or exclusion criteria or as study endpoints in clinical research (e.g., [14], pp. 108–109; [40], pp. 22–23; [41], pp. 39–41; [42] pp. 9–17; pp. 24–28), and our results suggest that this practice may be less strongly supported than is currently assumed (although see [28]). While not our primary goal, our results suggest that intra-individual variability (IIV), based on MMSE items, *increases* with greater levels of dementia severity. This comports with other published work using other tasks (e.g., [43]–[46]). Whether our results reflect IIV or not, they suggest that “point loss” may be an inappropriate proxy for “cognitive decline” with tests like the MMSE.

When measurement error is *not* independent of the true score, then estimating reliability for the set of items as a whole becomes considerably more complicated (see [3] for CTT-based estimation of reliability when error and true score are not independent; see [28] for discussion of reliability in longitudinal assessments; see also [47]). If our results are borne out with independent samples and other, less-restrictive (but still empirical) definitions of measurement error, reliability should not be estimated by CTT for tests like the MMSE.

## Supporting Information

Appendix S1 Model fitting details and estimation of DI and π*.
(DOC)
