# Supplementary material for: Using the Guttman Scale to Define and Estimate Measurement Error in Items over Time: The Case of Cognitive Decline and the Meaning of “Points Lost”
Source: PLoS One. 2012 Feb 17;7(2):e30019. doi: 10.1371/journal.pone.0030019 (PMC3281811; doi:10.1371/journal.pone.0030019)
Supplement: Appendix S1 — Model fitting details and estimation of DI and π*. (DOC) [file pone.0030019.s001.doc]

Appendix S1. Model fitting details and estimation of DI and π*.

A five-class restricted latent class model was fit to the data. The five classes represent the five valid response vectors for the Guttman model (i.e. 1111, 1110, 1100, 1000, 0000 as shown in Table 1). In these vectors, 1 indicates a correct response, with probability, and 0 indicates an incorrect response, with probability. The response probability,, was assumed to be constant across all latent classes, items, and time points. One model fit index, the Dissimilarity Index (DI), was computed based on *V*=four dichotomous (right =1, wrong=0) variables per item. The formula for DI (given by [23 p. 20]) is:

, where N= total number of cases, *n_s_* = observed frequency and = expected frequency for the *s*th response (*s*=16 in this case) vector. The expected frequency is a function of N and the estimated probability of that response vector (). Starting values for these probabilities were the observed frequency values.

The other model fit index, π*, was estimated based on a four-step procedure described in Dayton (2003) [23]. This method utilized a non-linear programming algorithm (NLP) implemented in Microsoft Excel Solver ([www.solver.com](http://www.solver.com/) for more information), originally proposed by [48,49]. NLP was shown to produce identical estimates of π* to those derived from the EM algorithm used by [50] who originally defined this index. The specifications on four steps used for NLP estimation using the Excel solver are outlined in [23] and briefly described here.

Specifications of NLP estimation of π*

1: Starting values for parameters.

The response probability for a correct response on an item, , was given the starting value of 1, and this was the start value across all four time points for each item.

2: Define expected frequency.

The expected frequency was computed for all possible scoring patterns with the initial , where counts were incremented only for patterns consistent with the 5-class Gutman model.

3. Impose the restriction or constraints.

The expected counts were constrained to be smaller than, or equal to, the observed count. The probability of a correct response, , was constrained to fall between zero and one. The expected proportion of individuals in each of the five possible latent classes was constrained to fall between zero and one. The sum of expected proportions across the five latent classes was one.

4: Define the objective function (π*) to be maximized as the sum of the expected frequency divided by the total number of observations.

Maximize π* = 1 – (Sum of expected frequencies across all response vectors)/(Total observed count).
